# Chemokine Signaling in Chemotherapy-Induced Neuropathic Pain

**DOI:** 10.3390/ijms20122904

**Published:** 2019-06-14

**Authors:** Laura Brandolini, Michele d’Angelo, Andrea Antonosante, Annamaria Cimini, Marcello Allegretti

**Affiliations:** 1 Dompé Farmaceutici SpA, Via Campo di Pile, 67100 L’Aquila, Italy; laura.brandolini@dompe.com; 2 Department of Life, Health and Environmental Sciences, University of L’Aquila, 67100 L’Aquila, Italy; dangelo-michele@hotmail.com (M.d.); andrea.antonosante@gmail.com (A.A.); 3 Sbarro Institute for Cancer Research and Molecular Medicine and Center for Biotechnology, Temple University, Philadelphia, PA 19122, USA

**Keywords:** chemotherapy, peripheral nervous system, central nervous system, inflammatory mediators, cytokines, chemokines

## Abstract

Chemotherapy-induced peripheral neuropathy (CIPN) is a side effect of chemotherapics such as taxanes, vinca alkaloids, and platinum compounds. In recent years, several reports have indicated the involvement of different molecular mechanisms in CIPN. The pathways described so far are diverse and target various components of the peripheral Nervous System (PNS). Among the contributors to neuropathic pain, inflammation has been indicated as a powerful driver of CIPN. Several pieces of evidence have demonstrated a chemotherapy-induced increase in peripheral pro-inflammatory cytokines and a strong correlation with peripheral neuropathy. At present, there are not adequate strategies to prevent CIPN, although there are drugs for treating CIPN, such as duloxetine, that have displayed a moderate effect on CIPN. In this review, we focus on the players involved in CIPN with a particular emphasis on chemokine signaling.

## 1. Introduction

Chemotherapy-induced peripheral neuropathy (CIPN) is a side effect of chemotherapics, such as taxanes, vinca alkaloids, and platinum compounds. Sensory neuropathy causes symptoms such as pain, allodynia, loss of sensation, paresthesia, numbness, tingling, and gait disturbance [1]. CIPN can result in significant loss of functional abilities and negatively impact quality of life, leading to lowering of the dose and discontinuation of assumption, and ultimately affecting overall survival rates [2]. Some chemotherapeutic drugs have been associated with a higher prevalence and duration of CIPN, such as taxanes and oxaliplatin treatment, which can last up to six months or two years after chemotherapy [1].

**Figure 1:**
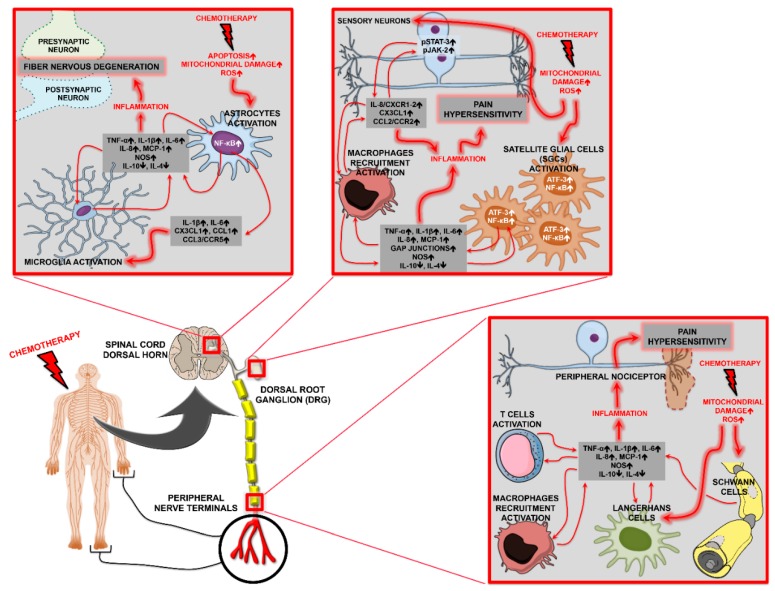
Summary scheme indicating the different players driving chemotherapy-induced peripheral neuropathy (CIPN).

In recent years, several reports have indicated the involvement of different molecular mechanisms in CIPN (Figure 1) [1]. The mechanisms described so far are diverse and target various components of the PNS. The Dorsal Root ganglion (DRG), which lacks an efficient blood–brain barrier (BBB) [3], is prone to neurotoxic damage and can account for the sensory symptoms seen in CIPN. Pt compounds trigger DNA damage through Pt adducts and cause changes in the nucleoli of DRG sensory neurons, affecting the transcription machinery [4]. The accumulation of taxanes and vinca alkaloids in the DRG seems to produce nucleolar abnormalities [5] and modifications in the neurofilaments [6]. They also affect microtubule conformation through tubulin acetylation (Figure 1) [7]. Bortezomib (BTZ) [8] and vinca alkaloids [9] modify axonal transport by decreasing the supply of trophic factors and energy production, or by increasing Wallerian-degeneration and causing neurological damage, which is often permanent. Energy depletion in axons due to mitochondrial damage may contribute to the neurotoxicity exerted by different chemotherapics [10,11,12]. BTZ affects the integrity of the endoplasmic reticulum, mainly in Schwann cells [8], thus causing degeneration of the myelin sheath. The modulation of axonal ion channels may also be involved in CIPN. Dysfunction in Na^+^ channels, mediated mainly by oxaliplatin, but also by paclitaxel and vincristine, can lead to an increase in Na^+^ currents in the DRG, predisposing it to paresthesia [13,14,15]. Moreover, Ca^2+^ and K^+^ channels are related to paclitaxel [16] and oxaliplatin toxicity [17], respectively. In addition, alterations in proteins involved in Ca^2+^ signaling (such as calpains and caspases) lead to apoptotic phenomena in the DRG [18]. Changes in the expression levels of transient receptor potential channels (TRPV, TRPA, and TRPM), as well as in molecules related to glutamate signaling induced by Pt compounds, resulting from treatment with paclitaxel and BTZ [19,20,21,22,23], lead to hyper-responsiveness of nociceptors, rendering patients prone to neuropathic pain and peripheral neuropathy development. Chemotherapics also induce increased expression of mitogen-activated protein kinases (MAPKs), leading to neurotoxicity [24]. Vincristine, paclitaxel, and BTZ cause inflammation due to an increase in pro-inflammatory cytokines in the peripheral nerves and the number of antigen presenting cells in the skin [16,25]. Furthermore, the production of reactive oxygen species (ROS), combined with an increase in Ca^2+^ in the DRG, is a common following chemotherapy and leads to neuronal cytotoxicity [26,27,28].

Among the players in neuropathic pain, inflammation has been indicated as a potential common driver of CIPN. Several pieces of evidence have demonstrated a chemotherapy-induced increase in peripheral pro-inflammatory cytokines and a strong correlation with peripheral neuropathy [29,30]. At present, there is no adequate strategy to prevent CIPN, although there are active drugs for treating CIPN, such as duloxetine, that have displayed a moderate effect on CIPN.

In this review, we focus on the players involved in CIPN with a particular emphasis on chemokine signaling. The pivotal mechanisms are summarized in Figure 1.

## 2. Chemotherapy-Induced Peripheral Neuropathy

CIPN is a dose-limiting side effect of chemotherapy that affects 30–40% of patients undergoing treatment [31]. It has been described as a functional impairment of neurons characterized by oxidative stress, inflammation, apoptosis, and electrophysiological failure.

It is generally accepted that, at the neuronal level, chemotherapeutic drugs damage microtubules and affect microtubule-based axonal transport, damage mitochondrial function, alter ionic homeostasis, or directly target DNA [32], leading to peripheral nerve degeneration or small fiber neuropathy. Taxanes and vinca alkaloids exhibit an antiproliferative effect by disrupting mitotic spindles and causing cell cycle arrest [32]. Platinum agents are known to cause CIPN by damaging the DRG through mitochondrial dysfunction and apoptosis, while also causing DNA damage or oxidative stress [33]. New drugs, such as bortezomib, eribulin, and ixabepilone, are also correlated with significant incidences of CIPN by affecting tubulin polymerization [33,34]. Glial cells seem to play a crucial role in CIPN. Alterations of Schwann cells, satellite cells in the DRG, and astrocytes in the spinal cord after chemotherapy lead to the activation of apoptosis [35]. Loss of glial cells results in a decrease in the protection and sustainment of nerve fibers and consequent defects in the propagation of the action potential [36]. Numerous findings indicate that CIPN, in addition to causing morphological changes, triggers the involvement of the inflammation and immune responses. Chemotherapy can cause mitochondrial DNA adducts and defects in electron transport chain proteins, leading to mitochondrial dysfunction [37,38]. This event is accompanied by disequilibrium in the redox potential and an increase in ROS within cells [37]. These reactive species can trigger perturbations in peripheral neurons, such as mitochondrial apoptosis, inflammation, and subsequent nerve degeneration [37,38]. ROS can also damage biomolecules such as phospholipids, resulting in demyelination, oxidized proteins, and an increase in carbonyl by-products, which can activate transient receptor potential vanilloid (TRPV) channels, impair antioxidant enzymes, and destroy microtubules [37]. Adducts to nuclear DNA and peroxynitrite create strand breaks, promoting neuronal apoptosis [39,40]. Intracellular ROS can also cause peripheral nociceptor over-excitation by increasing pro-inflammatory mediators (interleukin (IL)-1β, tumor necrosis factor-α (TNF-α), bradykinin, and nerve growth factors) [37,41]. All these metabolic, bioenergetic, and functional impairments lead to the development and maintenance of peripheral neuropathic injuries in neurons [37]. On the basis of these observations, it appears that preventative therapies for CIPN are urgently required for patients receiving chemotherapy.

Although many strategies have been developed, no specific intervention is presently recommended for the prevention or management of CIPN. In many patients, chemotherapy is discontinued due to CIPN, which increases the risk for patients. No efficient treatment options are presently available for CIPN because its exact pathophysiological mechanisms are not yet fully elucidated. Most of the pharmacologic treatments available for neuropathic pain include tricyclic antidepressants and anticonvulsants, which are minimally effective for CIPN and/or have substantial side effects [42,43,44]. At present, only duloxetine is recommended by the American Society of Clinical Oncology (ASCO) for CIPN treatment, based on a modest positive result obtained in one randomized controlled trial (RCT) [45].

## 3. Chemotherapy and the Immune System

Chemotherapy significantly affects the immune system. The effect is generally immunosuppressive due to the cytotoxic activity of chemotherapy on dividing immune cells. Other findings, however, have proposed that some chemotherapeutics may stimulate the immune system [46,47]. In breast cancer patients, taxanes increase the serum concentration of pro-inflammatory cytokines and increase peripheral blood natural killer and lymphocyte-activated cytotoxic cells [48]. In some individuals, acute immune responses occur, generally upon oxaliplatin treatment, leading to an increase in pro-inflammatory cytokines [49]. The immune response induced by chemotherapeutics varies greatly. This is particularly important in the clinical setting, since the tumor itself may determine immunomodulation, and the anti-tumor effects of chemotherapy often depend on the function of the immune system [50]. Several reports have shown an increase in the neuroimmune response following chemotherapy in animals. Paclitaxel causes an elevation in macrophage activity in peripheral nerves and the DRG. These macrophages are hypertrophic, which is indicative of an activated phenotype [46].

Macrophage activity in the DRG is increased in mice models of neuropathy induced by different chemotherapics [46]. The activation of macrophages is one of the causes of CIPN, since inhibition of their activation, with minocycline and clodronate, or with antibodies blocking chemokine (C-C motif) ligand 2 (CCL2), ameliorated pain hypersensitivity [47]. Paclitaxel and vincristine induce Langerhans cells in the epidermis of rodent paws, as indicated by an increase of positivity for protein 9.5 (PGP9.5) [47]. Activated Langerhans cells may cause neuropathic pain by augmenting nitric oxide and pro-inflammatory cytokines [47]. Generally, animal models show CIPN and an increased pro-inflammatory response in the PNS following treatment with different chemotherapeutics, such as taxanes, platinum-based drugs, and vinca alkaloids. Drugs able to block the activation of the innate immune response ameliorated pain hypersensitivity. However, it remains unclear if the direct cause of CIPN is the chemotherapy-associated neurotoxicity, which is increased by immune activation, or the immune activation itself.

## 4. Chemotherapy, Glia, and Neurons

The PNS contains distinct glial cells. Neurons in the DRG are endowed with satellite glial cells (SGCs), while axons are covered by Schwann cells. Schwann cells and SGCs change their phenotype following chemotherapy and secrete mediators that increase neuronal excitability, leading to pain hypersensitivity [8,51]. After nerve injury, Schwann cells also produce pro-inflammatory cytokines, such as IL-6 [52], adenosine triphosphate (ATP) [53], and chemokines, which sustain neuroinflammation through macrophage recruitment [54]. In experimental CIPN, Schwann cells express activating transcription factor-3 (ATF-3). High levels of ATF-3 and mitochondrial and endoplasmic reticulum modifications were reported in SGCs following chemotherapy [51,53,55]. Furthermore, a significant increase in glial fibrillary acidic protein (GFAP) levels and an increase in gap junction-mediated coupling were observed during paclitaxel and oxaliplatin treatments. Inhibitors of gap junctions improved pain and decreased SGC coupling [55]. In the same cells, paclitaxel caused a decrease in phosphoglycerate dehydrogenase, the enzyme responsible for the biosynthesis of L-serine. L-serine administration ameliorated both pain sensitivity and deficits in sciatic nerve conduction [56]. Microglia and astrocytes are involved in neuropathic pain as they release pro-inflammatory mediators [57,58]. Central nervous system glial cells secrete pro- or anti-inflammatory cytokines and chemokines, ATP, and other inflammatory factors which can affect neuronal activity [59,60]. It is generally accepted that long-term activation of spinal astrocyte during CIPN exists. However, transient activation [61], significant increases in activation [62], or no change in activated spinal microglial cells [63,64,65] have also been reported. It has been reported that paclitaxel causes the activation of astrocytes in the spinal cord dorsal horn [62,65]. This event is also observed in the absence of microglial activation—occurring only 4 h after paclitaxel administration and lasting for 28 days [65]. Guida et al. reported that communication with glia and microglia is necessary for the activity of dorsal horn neurons. The authors describe the anti-nociceptive effect of PC1, a non-peptide PKR1 (prokineticin 1) antagonist, in a mouse model of neuropathic pain. PC1 treatment significantly reduced the development of pain by decreasing spinal microglial and glial activation. PC1 reduced pain behavior and spinal neuronal sensitization in neuropathic mice [66]. Similarly, Luongo et al. demonstrated that allodynia was accompanied by an increase in microglial cells in the spinal cord using a model of Guillain–Barré syndrome. The expression of C-X3-C motif ligand 1 (CX3CL1, fractalkine) and its receptor CX3CR1 was increased in the Guillain–Barré model of dorsal horns, indicating a role for spinal microglia and CX3CL1/CX3CR1 in pain behavior [67]. Spinal changes were associated with mechanical hypersensitivity in a model of Guillain–Barré syndrome [67]. Finally, minocycline, a known microglial inhibitor, decreased astrocyte activation and reduced mechanical allodynia, indicating a crucial role for astrocytes in CIPN [68]. Moreover, several findings on microglia activation following paclitaxel treatment have also been reported [25,69]. Cannabinoid agonists or minocycline treatments decreased microglia activation as well as both mechanical allodynia and pro-inflammatory cytokine production [62].

## 5. Cytokine Signaling in CIPN

The inflammatory response triggered by chemotherapeutics has been indicated as a possible driver of the nociceptive process in CIPN [70,71]. The release of pro-inflammatory and chemotactic cytokines (chemokines) upon treatment has been suggested to be one of the primary mechanisms regulating neuro–immune interactions. Downstream cytokine effects are pivotal triggers of neuroinflammation in the sensory nervous system [72,73]. Chemotherapeutic administration significantly increases the production and release of cytokines, such as TNF-α, IL-1β, and IL-6, and chemokines, such as IL-8 and MCP-1 [25,74,75]. Pro-inflammatory cytokines may be responsible for neural cytotoxicity, not only through inflammation but also through direct activity, mediated by specific receptors, on neurons and glial cells [76,77,78,79]. Several preclinical observations have indicated the involvement of cytokine signaling in the pathogenesis of CIPN. Several studies have indicated an increase in pro-inflammatory cytokines (TNF-α, IL-1β, and IL-6), combined with a decrease in anti-inflammatory cytokines in the DRG and spinal cord [25,40,63]. The role of inflammation in vincristine-induced PN is still debated. An increase in Langerhans cells (LCs) in the skin, which leads to intraepidermal nerve fiber loss, has been reported as a consequence of inflammatory mechanisms [16]. The onset of pain triggered by an increase in LCs has been attributed to two main events: an increase in nitric oxide release [80] and the release of pro-inflammatory cytokines and neurotrophic factors [81,82], both of which result in the sensitization of nociceptors and mechanical hypersensitivity. In support of these findings, it has been shown that spironolactone, an aldosterone receptor antagonist with anti-inflammatory properties, has a beneficial effect in improving vincristine-related pain [71].

Several findings suggest that several inflammation processes (the increase of LCs, the regulation of pro-inflammatory cytokines, macrophage accumulation, and microglia activation) are involved in the onset of neuropathic pain following chronic treatment with paclitaxel. As mentioned previously, an increase in LCs in paclitaxel-treated rat skin has indicated the involvement of these cells in the development of pain [16]. LCs trigger pain development by inducing the release of nitric oxide [80], neurotrophic factors [82], and pro-inflammatory cytokines [81], resulting in the sensitization of nociceptors and leading to mechano-hypersensitivity. Several findings suggest that pro-inflammatory cytokines, such as IL-1 and TNF-α, are critical in increasing paclitaxel-induced neuropathic pain. Upregulation of pro-inflammatory cytokine gene expression in lumbar DRG following paclitaxel treatment has been reported [25]. Furthermore, an initial upregulation of ATF-3 in the DRG and Schwann cells, followed by macrophage activation in the DRG and sciatic nerve and microglial and astrocyte activation in the spinal cord, has been described [51]. Paclitaxel increased TNF-α and IL-1β and decreased IL-10 and IL-4 in the spinal cord, in association with peroxynitrite elevation due to the increased activity of nitric oxide synthase and nicotinamide adenine dinucleotide phosphate oxidase. Inhibition of peroxynitrite formation sharply decreased TNF-α and IL-1β and augmented IL-10 and IL-4 expression [40,47]. Another report indicated that IL-6 and soluble IL-6R (sIL-6R) levels were increased after chemotherapy in breast cancer patients reporting CIPN as compared with those without CIPN [83]. Paclitaxel also activates the ceramide-sphingosine 1- phosphate pathway, blocks sphingosine 1-phosphate receptor subtype 1 (S1PR1), ameliorated paclitaxel-induced CIPN, and decreased TNF-α and IL-1β production while increasing IL-4 and IL-10 release in the spinal cord [63]. This was recently confirmed by Cheng et al., who reported that reduced sphingosine-1-phosphate (S1P) in the spinal cord in response to nerve injury causes neuropathic pain by activating S1PR1 in astrocytes. Genetic and pharmacological inhibition of S1PR1 by selective antagonists in distinct chemical classes decreased and counteracted neuropathic pain in mice models of traumatic nerve injury. The antagonists maintained the capability to inhibit neuropathic pain during sustained drug treatment, and these effects were independent of opioid circuits. Moreover, knockouts of S1PR1 in mice astrocytes led to the absence of neuropathic pain following nerve injury, indicating astrocytes as the primary inducer of S1PR1 activity. At the molecular level, it has been demonstrated that the decrease in neuropathic pain caused by S1PR1 inhibition was due to IL-10, an anti-inflammatory cytokine [84].

Additionally, elevated levels of IL-1β in paclitaxel-treated rats have been associated with glycogen synthase kinase 3β (GSK3β) activation. Inhibition of GSK3β activity counteracted pain hypersensitivity and IL-1β release in the dorsal spinal cord [85]. It has been reported that augmented expression of TNF-α, IL-1β, and IL-6 in the spinal cord following paclitaxel treatment lasted for eight days and was no longer present at day 29, indicating a transient increase in cytokine production in the Central Nervous System (CNS) [62].

In the PNS, elevated expression of IL-1β and TNF-α was reported in the DRG of animals after 36 days of paclitaxel treatment and was decreased by IL-10 gene therapy [25,47]. Moreover, it has been established, in vitro and in vivo, that sensitive neurons are able to modulate cytokine production, thus contributing to the onset of CIPN [86]. Oxaliplatin treatment in rats caused an increase in IL-1β and TNF-α and a decrease in IL-10 and IL-4 in the spinal cord after 25 days of treatment [63].

Altogether, these findings indicate that pro-inflammatory cytokine expression is generally upregulated in both the CNS and PNS following chemotherapy treatment in animal models.

## 6. Affective Disorders and Neuropathic Pain

Debilitating symptoms, including allodynia, hyperalgesia, and neuropathic pain, have a strong negative impact on patients’ quality of life. The role of the immune system in altered sensation following nerve injury is well known. However, its role in the development of affective-motivational disorders remains mostly unknown. The elevation of pro-inflammatory cytokines described in pain conditions is also evident in the “sickness response”, which involves a series of inflammatory events in response to pathology and encompasses a broad range of physiological and behavioral aspects, such as neuropathic pain. It is worth noting that these response to illness are quite similar to some specific features of the depressed status in affective disorder [87]. Similarly, Austin et al. demonstrated that the immune system plays an individual-specific role in the different behaviors observed after nerve injury, in some cases resulting in affective-motivational impairment [88]. A study reported on the effect of the microglial inhibitor minocycline on depressive behavior and mechanical and cold allodynia induced by spinal nerve ligation as well as the associated modulation of genes encoding for microglial markers and inflammatory mediators in the prefrontal cortex of a rat model of depression [89]. Minocycline treatment decreased the marker of microglial activation and the pro-inflammatory cytokine IL-1β. In parallel, the M2 microglia marker and anti-inflammatory cytokine IL-10 were augmented by minocycline administration. Thus, decreased microglia activation reduced neuropathic pain behavior and the levels of inflammatory mediators, depending on the presence or absence of a depressive phenotype. Other studies have indicated that that anxiety and chronic pain are concomitant, but neural substrates for their comorbidity are unclear [90].

However, even if the increase in pro-inflammatory cytokines and chemokines and microglia activation are common events in chronic pain and affective/depressive disorders, the neural substrates for their comorbidity are unclear.

## 7. Chemokine Signaling in CIPN

Chemokines play a critical role in the activation and infiltration of macrophages and glial cells in several neuropathic pain conditions [57,73,91]. Chemotherapy induces an upregulation of the expression of chemokines, including CCL2 and CX3CL1, in sensory neurons [92,93,94,95]. The importance of the CCL2/CC chemokine receptor type 2 (CCR2) signaling pathway is well established in paclitaxel animal models of CIPN, whereby CCL2/CCR2-mediated signaling is linked to the recruitment and activation of monocytic cells and the development of pain hypersensitivity [94,96,97]. Paclitaxel is known to activate toll-like receptor (TLR)-4 signaling in rodents, which mimics molecular damage [96,98,99]. This has been suggested to be the mechanism that induces increased expression of CCL2, thereby promoting macrophage infiltration of the DRG in CIPN [100]. CX3CL1 is upregulated predominantly in A-fiber sensory neurons in the DRG following paclitaxel treatment, and blocking signaling using a CX3CL1-neutralizing antibody has been shown to inhibit macrophage infiltration and lead to neuronal apoptosis and mechanical allodynia [101]. CX3CL1 also activates CX3CL1R monocytes in the sciatic nerves of vincristine-treated mice, causing an increase in ROS and TRPA1 channels in the sensory nerves involved in pain [47]. Among the chemokines, IL-8 and its receptors CXCR1/2 are upregulated in several animal models of nerve injury and play a key role in the development of neuropathic pain and hypernociception [102,103]. In a recent study in patients with peripheral neuropathy, IL-6 and IL-8 expression appeared to be sharply increased in skin biopsies [104], drawing attention to IL-8 and IL-6 as promising pharmacological targets for pain management. In our previous work, we demonstrated that IL-8 CXCL8 signaling was able to modulate cellular biomarkers of pain in sensory neurons (i.e., p-STA3 and p-JAK2) by activating its specific receptors CXCR1 and CXCR2 [86]. In the CNS, paclitaxel causes a significant increase in the chemokine CCL3 and its receptor CCR5 in DRG neurons, with the appearance of activated microglia also being observed. In parallel, there was a large elevation in the purinoceptor P2X7, which modulates the secretion of CCL3 [105]. In the same way, paclitaxel increases CX3CL1 in the spinal cord with concomitant increase in CCL1, IL-6, IL-1β, and IL-15 mRNA expression, accompanied by microglia activation [106]. Paclitaxel was also responsible for increasing CCL2 expression in the spinal cord. CCL2 expression was strongly decreased by cannabinoid receptor type 2 (CB2 -targeted agonist (AM1710) administration [81].

A recent exhaustive review of the literature identified genes implicated in CIPN and used Ingenuity Pathway Analysis (IPA) bioinformatics tools to depict comprehensive pathway and network analyses of these genes [107]. The IPA core analysis for the genes associated with CIPN showed that *IL6*, *TNFα*, *CXCL8*, *IL1β*, and *ERK1/2* are key genes in terms of the number of connections, suggesting either direct or indirect interrelations with nervous tissue, thus leading to CIPN following chemotherapy. Interestingly, some studies in cancer patients have indicated that cytokine gene polymorphisms [108,109,110,111,112,113,114,115,116], such as *IL6*, *TNFα*, and *IL1β,* may play a role in pain. These findings suggested that cytokine secretion or tissue damage modulates the activity of nociceptors, contributing to pain hypersensitivity.

Further evidence is required to confirm the causal relationship between systemic cytokine and chemokine levels following chemotherapy and the development of CIPN symptoms.

The effects of chemokines on neuropathic pain are complex, and further efforts are needed to elucidate their role in cancer-derived pain. It is therefore important to identify selective chemokine inhibitors of clinical grade, which would allow for an understanding of the therapeutic potential of this approach.
